# The Association Between Loneliness and Inflammation: Findings From an Older Adult Sample

**DOI:** 10.3389/fnbeh.2021.801746

**Published:** 2022-01-11

**Authors:** Karina Van Bogart, Christopher G. Engeland, Martin J. Sliwinski, Karra D. Harrington, Erik L. Knight, Ruixue Zhaoyang, Stacey B. Scott, Jennifer E. Graham-Engeland

**Affiliations:** ^1^Department of Biobehavioral Health, The Pennsylvania State University, University Park, PA, United States; ^2^Ross and Carol Nese College of Nursing, The Pennsylvania State University, University Park, PA, United States; ^3^Department of Human Development and Family Studies, The Pennsylvania State University, University Park, PA, United States; ^4^Center for Healthy Aging, The Pennsylvania State University, University Park, PA, United States; ^5^Department of Psychology and Neuroscience, University of Colorado Boulder, Boulder, CO, United States; ^6^Department of Psychology, Stony Brook University, Stony Brook, NY, United States

**Keywords:** loneliness, inflammation, C-reactive protein (CRP), older adults, ecological momentary assessment (EMA), psychoneuroimmunology

## Abstract

Loneliness has been linked to poor mental and physical health outcomes. Past research suggests that inflammation is a potential pathway linking loneliness and health, but little is known about how loneliness assessed in daily life links with inflammation, or about linkages between loneliness and inflammation among older adults specifically. As part of a larger investigation, we examined the cross-sectional associations between loneliness and a panel of both basal and LPS-stimulated inflammatory markers. Participants were 222 socioeconomically and racially diverse older adults (aged 70–90 years; 38% Black; 13% Hispanic) systematically recruited from the Bronx, NY. Loneliness was measured in two ways, with a retrospective trait measure (the UCLA Three Item Loneliness Scale) and an aggregated momentary measure assessed *via* ecological momentary assessment (EMA) across 14 days. Inflammatory markers included both basal levels of C-reactive protein (CRP) and cytokines (IL-1β, IL-4, IL-6, IL-8, IL-10, TNF-α) and LPS-stimulated levels of the same cytokines. Multiple regression analyses controlled for age, body-mass index, race, and depressive symptoms. Moderation by gender and race were also explored. Both higher trait loneliness and aggregated momentary measures of loneliness were associated with higher levels of CRP (β = 0.16, *p* = 0.02; β = 0.15, *p* = 0.03, respectively). There were no significant associations between loneliness and basal or stimulated cytokines and neither gender nor race were significant moderators. Results extend prior research linking loneliness with systemic inflammation in several ways, including by examining this connection among a sample of older adults and using a measure of aggregated momentary loneliness.

## Introduction

By nature, humans are social beings and possess a fundamental need to feel connected to others. When this need is not met, feelings of loneliness arise. Loneliness is defined as a subjective, unpleasant psychological state of feeling alone that stems from a discrepancy between desired and actual social relationships (Peplau and Perlman, 1982). Chronic feelings of loneliness are robustly linked with the development of negative health outcomes, including depression, anxiety (Meltzer et al., 2013), suicidal ideation and behavior (Rudatsikira et al., 2007), cardiovascular disease (Holt-Lunstad and Smith, 2016), and all-cause mortality (Patterson and Veenstra, 2010; Rico-Uribe et al., 2018). Loneliness often predicts health separately from objective measures of social isolation (Cacioppo et al., 2015; O'Súilleabháin et al., 2019), highlighting the importance of the subjective experience of loneliness. While the mechanisms underlying the link between loneliness and health are not fully understood, growing evidence suggests that inflammation may play a significant role in this relationship (Hawkley and Cacioppo, 2003; Hawkley et al., 2007; Kiecolt-Glaser et al., 2010; Cacioppo and Cacioppo, 2018).

Inflammation is the body's natural response to physical injury (e.g., a cut or other injury), and is a biological response that protects against infection as well as aids in healing (Engeland and Marucha, 2009; Engeland and Gajendrareddy, 2011). From an evolutionary perspective it makes sense that inflammation increases during moments of psychological stress, given that stress often co-occurs with physical harm (Cacioppo and Cacioppo, 2018). Although exposure to modern psychological and social stress (such as loneliness) does not pose a direct threat through physical injury, it nonetheless triggers activation of the hypothalamic-pituitary-adrenal (HPA) axis and autonomic nervous system (Hänsel et al., 2010; Hennessy et al., 2014; Eisenberger et al., 2017). Repeated over-activation of such processes can contribute to immune dysregulation, including the development of chronic low-grade inflammation (Segerstrom and Miller, 2004; Kiecolt-Glaser et al., 2010), which has been linked to cardiovascular disease (Arenillas et al., 2003; Kivimäki and Steptoe, 2018), Alzheimer's disease (Heneka and O'Banion, 2007), and cancer (Schetter et al., 2010), among many other negative health outcomes. Such phenomena underlie the Evolutionary Theory of Loneliness (Cacioppo and Cacioppo, 2018), which proposes that stress-induced immune dysregulation is responsible for the connection between loneliness and inflammation.

Lonelier individuals tend to exhibit greater inflammatory responses to acute psychological stress, a phenomenon replicated across several studies (for review see Brown et al., 2018). Further, it has been established that loneliness is associated with higher pro-inflammatory gene expression (Cole et al., 2007), indicating an upregulation of inflammatory signaling that can be a precursor for higher systemic inflammation (Irwin and Cole, 2011; Ligthart et al., 2018) and worse health (Slavich and Cole, 2013; Simons et al., 2017). However, findings from studies linking loneliness to systemic, circulating inflammatory markers are less consistent. Some studies have reported significant associations between loneliness and higher circulating (basal) levels of inflammatory markers (Nersesian et al., 2018; Zilioli and Jiang, 2021), but there are also null findings in this literature (Hackett et al., 2012; Mezuk et al., 2016; Zilioli and Jiang, 2021). A recent meta-analysis found that a significant association between loneliness and circulating IL-6 levels has been observed consistently, whereas associations between loneliness and C-reactive protein (CRP) or fibrinogen have not been consistent (Smith et al., 2020). In longitudinal work, however, some studies have reported significant associations between trait loneliness and CRP among different age samples [50–99 years: (Vingeliene et al., 2019) and 50–67 years: (Cole et al., 2007)]. These inconsistent findings with regard to how loneliness relates to various cytokines and CRP suggest more work is needed to differentiate the unique impact that loneliness has on inflammatory load.

Even less is known about how loneliness relates to stimulated levels of inflammatory markers, which reflect the ability of circulating cells to respond to immunogenic challenge. One study showed that lonelier individuals exhibited increased TNF-α and IL-6 in response to endotoxin (*Escherichia coli*) injection (*in vivo*) (Moieni et al., 2015). Another study found that lonelier individuals showed higher lipopolysaccharide (LPS) stimulated IL-1β production in response to an acute psychological stressor than less lonely individuals (Jaremka et al., 2013). The link between stimulated cytokines and health risk is still emerging, but initial evidence suggests a positive association between higher stimulated cytokines and worse health. For example, one study found that patients with rheumatoid arthritis had higher levels of LPS-stimulated cytokines compared to healthy controls (Scuderi et al., 2003). Another study found that among post-myocardial infarction patients, those who went on to experience heart failure exhibited higher levels of LPS- stimulated cytokines than those who did not (Satoh et al., 2006). What is clear is that stimulated cytokines and peripheral circulating cytokines measure distinct measures of inflammation (Davis et al., 2020), with stimulated cytokines tapping into the magnitude of response from white blood cells to immunological challenge, vs. the relatively static measure of peripheral inflammation. Although both the degree and manner remain to be determined by which basal cytokines, stimulated cytokines, and CRP differentially predict health outcomes, it is important to assess a broad range of distinct inflammatory markers in order to build an initial evidence base linking loneliness to inflammation (and health).

Most of the previous work on loneliness has relied on retrospective trait measures, such as the UCLA Three Item Loneliness Scale (Hughes et al., 2004), a self-report measure that asks individuals to report how often they typically feel lonely. Although many such retrospective global measures show predictive validity for health outcomes (e.g., Luo et al., 2012; Perissinotto et al., 2012), these types of measures can be influenced by recall bias as well as broad perceptions of the self; as such, they may capture how individuals *think* they typically feel, not necessarily how they actually feel (Shiffman et al., 2008). Therefore, it may be useful to gather information about loneliness as it is experienced in everyday life. Ecological momentary assessment (EMA) is a method in which momentary levels of psychological states (such as loneliness) are repeatedly assessed in naturalistic environments, such as multiple times per day across days in the same individual while they go about their usual activities (Smyth et al., 2017). The average of these momentary levels may serve as an indicator of a person's typical or trait-like level of loneliness, as it reflects the amount of loneliness people have typically reported across a period of time (Moore et al., 2016). Previous research among older adults found that compared to retrospective trait measures, EMA derived measures of mindfulness, depression, and anxiety yielded larger effect sizes in response to an intervention (Moore et al., 2016). Additionally, in comparison to retrospective measures, multiple studies have found that momentary measures of psychosocial stress and emotions have better predicted physiological markers indicative of disease risk (for review, see Conner and Barrett, 2012), including inflammatory markers (Graham-Engeland et al., 2018). Given that loneliness fluctuates in daily life (van Roekel et al., 2014; Compernolle et al., 2021), an aggregated momentary measure of loneliness may better reflect *experienced* loneliness and may relate differently to inflammation as compared with global, retrospective assessments. We are unaware of other research that has examined how aggregated momentary measures of loneliness relate to inflammation.

Loneliness is common during older adulthood (Ong et al., 2016; Courtin and Knapp, 2017) and higher rates of mortality among lonelier older adults have been reported in a number of studies (Luo et al., 2012; Perissinotto et al., 2012; Luo and Waite, 2014). A recent comprehensive report was published by the National Academy of Sciences that highlights the impact of loneliness on older-adult health and the need for more research in this domain broadly (National Academies of Sciences, 2020). With inflammation increasing with age and older adults being at greater risk of developing inflammatory-related health conditions (Graham et al., 2006a), it is particularly important to investigate linkages between loneliness and inflammation among older adults. However, most studies examining the link between loneliness and inflammation have focused broadly on middle-aged adults. One longitudinal study of older adults (specifically, ages 61–70 years at the baseline timepoint of the study) found that the onset of loneliness was associated with an increase in levels of CRP (Vingeliene et al., 2019). Additionally, other work has found that among adults 70–79 years old, lower social integration (an objective measure of social network size) was associated with higher levels of fibrinogen (Loucks et al., 2005), CRP (Loucks et al., 2006a), and IL-6 (Loucks et al., 2006b). However, although social integration may relate to loneliness, social integration and loneliness are fundamentally different constructs, and there appear to be no studies that specifically examine the link between loneliness and inflammation in individuals aged 70 years and older.

Gender and race may affect the link between loneliness and inflammation. Gender differences in the levels and rates of loneliness have not been observed consistently, but there is some suggestion that loneliness may be more prevalent among older women because they tend to outlive their partners (Vozikaki et al., 2018). Although some studies have found no gender differences in the link between loneliness and inflammation (Steptoe et al., 2004; Mezuk et al., 2016), a recent meta-analysis (Maes et al., 2019) suggests that loneliness may be associated with worse health-related outcomes for women relative to men (Steptoe et al., 2004; Thurston and Kubzansky, 2009; Cao and Liu, 2020). Further, there are strong links between gender and inflammation, with women tending to have higher levels of circulating inflammatory markers, such as CRP (Darnall and Suarez, 2009; O'Connor et al., 2009).

In contrast to gender, the role of race in relation to loneliness and its link to inflammation is relatively unexplored. Extant literature links race (and racism) with health disparities and inflammatory markers (O'Connor et al., 2009; Ransome et al., 2018; Williams et al., 2019) and some research suggests that loneliness may be higher among Black vs. White older adults (Hawkley et al., 2019). Past research has found that older Black individuals exhibit more health disparities and are less likely to live with a spouse or partner (especially Black women), compared to White individuals (Hawkley et al., 2019). A better understanding of how gender and race relate to the link between loneliness and inflammation will help identify sub-populations most at risk of negative health effects of loneliness.

The goal of the present research was to examine the associations between self-reported loneliness and a broad panel of both basal and stimulated inflammatory markers among a diverse sample of older adults. Specifically, we test the associations between both retrospective trait loneliness and aggregated momentary loneliness with three sets of inflammatory markers: basal cytokines, stimulated cytokines, and CRP. We expected that higher levels of both retrospective trait loneliness and aggregated momentary loneliness would be associated with higher values of inflammatory markers. Additionally, we test whether gender and race moderate these associations. Based on previous research illustrating gender differences related to health outcomes, we hypothesized that the link between loneliness and inflammation would be stronger among women and we examined race as a moderator on an exploratory basis.

## Methods

### Participants

As reported elsewhere in more detail (Zhaoyang et al., 2021), systematic random sampling was used to recruit participants using Medicare and New York Registered Voter Lists in Bronx County data as part of the ongoing Einstein Aging Study (EAS). Data and blood samples from the current study were collected between May 2017 and March 2020 [i.e., prior to the announcement by the World Health Organization of the COVID-19 pandemic (March 11, 2020) and the U.S. government-issued stay-at-home orders]. Recruitment letters were mailed to explain the study goals and interested participants were screened for eligibility over the phone. Inclusion criteria for EAS included those who agreed to participate, were 70 years or older and ambulatory, fluent in English, and were residing in the community. The total available sample of participants who provided inflammation data was 296. For the current study, participants were excluded if complete loneliness or inflammation data were unavailable for them, or if they were taking strong anti-inflammatory or immunosuppressant medications. The final analytic sample (*N* = 222) had a mean age of 76.82 (*SD* = 4.72) and did not significantly differ in key sociodemographic factors (including age, gender, race, and ethnicity) from the larger sample (see Table 1 for additional sample characteristics).

**Table 1:** Participant characteristics (*N* = 222).

	**Mean or %**	**SD**
Sex/gender	63% (*n* = 139) women	
Age	76.82	4.72
Race/ethnicity		
White (non-Hispanic)	47% (*n* = 105)	
Black	37% (*n* = 83)	
Hispanic, White	11% (*n* = 24)	
Hispanic, Black	3% (*n* = 6)	
Asian	1% (*n* = 3)	
Income	41% (*n* = 91) < $30,000	
Education (years)	15.05	3.61
Married	35% (*n* = 77) married	
Live alone	53% (*n* = 117) live alone	
BMI	29.10	5.90
Number of chronic health conditions	2.29	1.36
Smoking status	3% smoke (*n* = 7)	
Loneliness (trait)	3.93	1.31
Loneliness (momentary aggregate)	13.03	16.57
PROMIS—depressive scores	11.15	4.55

*1 participant did not report income data; 1 participant did not report race/ethnicity*.

### Procedure

Following the phone screening assessment, eligible participants provided consent and completed questionnaires (prior to the EMA protocol) to assess demographic and psychosocial characteristics; this included assessment of retrospective trait loneliness. Next, participants received training on the EMA protocol (including how to use the study smartphones) during a visit at the research clinic. After this, participants undertook a 2-day practice session of the EMA protocol, followed by the 14-day formal EMA burst. The EMA protocol included five assessments of momentary loneliness from four quasi-randomly beeped surveys throughout the day and a self-initiated end-of-day-survey. Blood samples were collected in the research clinic; all participants included in the current analyses provided a fasting blood sample at the beginning and a non-fasting blood sample at the end of the 14-day EMA burst, at approximately the same time of day (morning).

### Measures

#### Loneliness

The Three-Item Loneliness Scale (Hughes et al., 2004) was used to assess trait loneliness prior to the start of the EMA protocol. Participants responded to three questions regarding their general frequency of lonely feelings on a 1 (hardly ever or never) to 3 (often) scale. A sum score was computed for each participant. Internal reliability for the three trait loneliness items in the present sample was α = 0.87. To assess momentary loneliness, participants responded to a single loneliness item that read “Do you feel lonely?” on a sliding scale bound by “not at all” to “extremely” (which was translated to a 0–100 scale); this was collected 5 times/day during the 14-day EMA burst described above. Before we aggregated these repeated measurements, we calculated the intraclass correlation (ICC) by fitting an empty multilevel model to the momentary loneliness variable. The ICC for momentary loneliness—the proportion of variability in momentary loneliness that may be attributed to stable, person level differences (relative to variation within persons across days)—was 0.70, suggesting it was appropriate to aggregate (Terwee et al., 2007). Person-means of the EMA loneliness responses were calculated and used in analyses to reflect an average momentary loneliness score.

#### Inflammation

Basal inflammatory markers were assessed from blood. Due to a protocol change early in the study^1^, 67 samples to be analyzed for cytokine levels were collected in heparin-coated tubes, with the remainder of samples collected in EDTA (ethylenediamine tetraacetic acid)-coated tubes; we determined that results reported here did not change when excluding samples collected with heparin-coated tubes, and all samples were retained^2^. All samples were centrifuged at 1,500 g for 15 min. The supernatant was aliquoted and stored at −80°C. Prior to centrifuge, stimulated cytokine levels were assessed from a 1 mL subsample of the same blood; this was incubated with LPS (1 μg/mL, *E. coli* 055:B5, Sigma Aldrich) on a rotational shaker at 37°C with 5% CO_2_ for 2 h. Samples were then centrifuged at 1,500 g for 15 min. Aliquots were made from supernatant and stored at −80°C. Basal cytokines (IL-1β, IL-4, IL-6, IL-8, IL-10, TNF-α), LPS-stimulated cytokines (IL-1β, IL-4, IL-6, IL-8, IL-10, TNF-α), and high sensitivity CRP were quantified using a multiplex (V-plex) assay (Meso Diagnostics, Rockville MD). This assay generally performs better than other common multiplex platforms (Belzeaux et al., 2017). All inflammatory markers that were measured are reported in the current analyses. The minimum detection limit for all cytokines (stimulated and basal) ranged between 0.02 and 0.07 pg/mL, and was 1.33 mg/L for CRP. All samples were run in duplicate. Sample pairs with coefficients of variation (CVs) <15% were rerun. Confirmed values below the minimum detection limit were replaced with zeros.

Pre- and post- EMA blood draw data were averaged to better capture inflammation across the full 2-week EMA burst. Given that all basal cytokines were significantly correlated (*r*'s ranging from 0.21 to 0.52), we performed an exploratory factor analysis to test whether these cytokines grouped together as a composite to represent overall inflammatory load. Results of this analysis revealed support for one factor (factor loadings for individual basal cytokines ranged from 0.50 to 0.66), similar to findings with past work that has taken the same approach (Graham-Engeland et al., 2018; Knight et al., 2020). Next, we ran an exploratory factor analysis to test whether the stimulated cytokines grouped together as one factor. Results of this analysis also revealed support for one factor (factor loadings for individual stimulated cytokines ranged from 0.69 to 0.91). Based on this, we created separate composite scores for both basal and stimulated cytokines by calculating the means of the z-scores for each cytokine. Both composite scores revealed good reliability (basal: α = 0.77; stimulated: α = 0.93). Our primary analyses for cytokines used these composite scores as outcomes; we also explored and report individual cytokine analysis as follow-up Supplementary Information.

#### Covariates

As prior research has found that age, body mass index (BMI) (Mohamed-Ali et al., 1997; Dentino et al., 1999), and race/ethnicity (Ranjit et al., 2007) tend to be associated with inflammatory markers (O'Connor et al., 2009), we included all of these in our final models. Additionally, because loneliness is often associated with depressive symptoms (Mezuk et al., 2016; Cacioppo and Cacioppo, 2018), which in turn is often associated with inflammation (Kiecolt-Glaser et al., 2015; Majd et al., 2018, 2020), we controlled for depressive symptoms in our final model to determine whether associations with loneliness were independent from depressive symptoms. Depressive symptoms were measured using the PROMIS Depression Short Form 8a, which contains eight items regarding how frequently a person feels depressed (PROMIS Health Organization, 2012). Due to past linkages with inflammation and/or loneliness, the following sociobehavioral and health-related factors were considered as additional possible covariates: education, income, marital status, living alone, current smoking status, and number of chronic health conditions. Only marital status, race, and current smoking status were associated with any inflammatory marker (see **Table 3**). Later analyses revealed that controlling for these variables did not change results; thus, to present parsimonious models, these variables were not included in the final models as covariates.

### Statistical Analysis

All models were estimated in R [version 4.0.4, (R Core Team, 2021)]. A value of *p* < 0.05 was considered statistically significant. Multiple linear regression analyses were conducted to determine the association between reported loneliness scores and inflammation levels. Six separate models were estimated: 1) trait loneliness predicting basal cytokine composite, 2) stimulated cytokine composite, and 3) CRP, and 4) aggregated momentary loneliness predicting basal cytokine, 5) stimulated cytokine composite, and 6) CRP^3^. To correct for skewness of inflammatory data, logarithmic (log) transformation was applied, using a log formula of (x+1) for all cytokines given the frequency of low values. In addition, to utilize data from the maximum number of participants while minimizing the influence of outliers, all inflammation data that were >3 standard deviations above the mean were winsorized to 3 standard deviations based on precedent (Graham-Engeland et al., 2018) and statistical recommendations (Tabachnick and Fidell, 2007); after removing one extreme outlier of CRP, no CRP values were >3 standard deviations above the mean, so only cytokine data were winsorized.

Moderation by gender was tested with a variable coded as 0 (women) or 1 (men). We tested the interaction by race with a variable coded as 0 (other) and 1 (White) (“other” consisted of 80% Black, 20% White Hispanic, 0.06% Black Hispanic, and 0.03% Asian). Because the “Other” category was not a homogenous group, we also tested the interaction by race with a variable coded as White/Black, excluding all other race categories.

## Results

### Descriptive Statistics

Participant characteristics are displayed in Table 1 and sample values of non-log-transformed inflammatory markers are displayed in Table 2. The study sample consisted of 222 adults ages 70–90 years old (Mean = 76.82, SD = 4.72; 63% women). The ethnic composition included 47% white (non-Hispanic), 37% Black, 11% Hispanic-white, 3% Hispanic-black, and 1% Asian/other. The average trait loneliness score for the sample was 3.93 (SD = 1.31), which is relatively low [a score between 6 and 9 is considered highly lonely (Hughes et al., 2004)]. The mean of the aggregated momentary loneliness scores for the sample was 13.03 (SD = 16.57), which is also quite low, given the range of possible scores (0–100). The correlation between the trait and aggregated momentary loneliness measures was *r*_(220)_ = 0.46 (*p* < 0.001). Average CRP levels for the overall sample were 4.30 mg/L (SD = 6.39). The mean CRP levels for individuals with higher vs. lower than average trait loneliness were 5.00 and 3.61 mg/L (*p* = 0.09), respectively; similar differences were evident between those with higher vs. lower aggregated momentary loneliness (5.78 and 3.66 mg/L; *p* = 0.07, respectively). Living alone was not significantly correlated with either measurement of loneliness (aggregated momentary or trait; *p*'s > 0.10). Although living alone was not significantly correlated with either cytokine composite score (*p*'s > 0.10) it was marginally correlated with CRP (*r* = 0.13, *p* = 0.053), meaning that individuals who live alone tended to have higher CRP levels. Bivariate associations between relevant study variables are displayed in Table 3.

**Table 2:** Means, medians, and standard deviations of non-log-transformed inflammatory markers.

		**Range**	**Median**	**Mean (SD)**
Basal cytokines (pg/mL)	IL-1β	0–1.87	0.08	0.10 (0.17)
	IL-4	0–0.55	0.01	0.02 (0.04)
	IL-6	0.18–190.68	0.93	2.32 (12.93)
	IL-8	1.00–50.25	3.95	4.83 (4.12)
	IL-10	0–1.60	0.21	0.27 (0.24)
	TNF-α	0.43–45.63	2.16	2.46 (3.06)
Stimulated cytokines (pg/mL)	IL-1β	0.07–669.22	18.55	38.51 (77.02)
	IL-4	0–5.6	0.17	0.38 (0.70)
	IL-6	0.71–1659.04	45.84	110.76 (227.28)
	IL-8	15.54–7429.44	117.73	428.10 (933.67)
	IL-10	0.09–16.64	0.49	1.10 (1.87)
	TNF-α	22.41–3279.68	279.47	403.91 (440.59)
CRP (mg/L)		0.19–53.72	2.44	4.30 (6.39)

**Table 3:** Bivariate associations between key participant characteristics and inflammatory markers.

**Participant characteristic**	**CRP**	**Basal composite**	**Stimulated composite**
Loneliness (trait)	*r* = 0.12	*r* = −0.08	*r* = 0.02
Loneliness (momentary aggregate)	*r* = 0.10	*r* = 0.05	*r* = 0.04
Sex/gender	***t*** **=** **2.23**	*t* = −0.15	*t* = −0.58
Number of chronic health conditions	*r* = 0.09	*r* = 0.17	*r* = −0.02
BMI	***r*** **=** **0.25**	*r* = 0.04	*r* = 0.01
Years of education	*r* = −0.03	*r* = −0.01	*r* = −0.05
Income	*t* = 0.24	*t* = 0.01	*t* = 0.61
Age	*r* = −0.05	***r*** **=** **0.16**	*r* = 0.06
Smoking status	***t*** **=** **−2.74**	*t* = −1.35	*t* = −0.71
Race	***t*** **=** **2.19**	*t* = −0.93	*t* = 0.33
Depressive symptoms	*r* = −0.06	*r* = 0.05	*r* = −0.09
Living alone	***t*** **=** **−1.94**	*t* = −1.13	*t* = −0.32
Marital status	***t*** **=** **2.20**	*t* = 1.62	*t* = 0.57

*Pearson correlations were computed for continuous variables and t-tests were used for dichotomous variables. Race was coded as Other Races = 0 and White = 1; Sex/gender was coded as female = 0 and male = 1; Marital status was coded as not married = 0 and married = 1; Income was coded as less than $30,000 = 0 and greater than $30,000 = 1; Bold indicates statistical significance at α = 0.05*.

### Basal and Stimulated Cytokines

No significant associations were found between basal cytokine composite scores and either trait loneliness (β = −0.11, *p* = 0.12) or aggregated momentary levels of loneliness (β = 0.01, *p* = 0.84) while controlling for age, race, BMI, and depressive symptoms. Full regression results are presented in Table 4. Similarly, no significant effects were found between stimulated cytokine composite scores and either trait loneliness (β = 0.08, *p* = 0.30) or aggregated momentary levels of loneliness (β = 0.06, *p* = 0.40) while controlling for age, race, BMI, and depressive symptoms (see Table 4). As shown in Supplementary Material, exploratory results with individual cytokines (both basal and stimulated) were not significantly associated with either loneliness measure.

**Table 4:** Results for main effects models.

		**Basal composite**	**Stimulated composite**	**CRP**
Trait	Intercept	**0.00 (0.02)**	0.00 (0.35)	0.00 (0.95)
	Trait loneliness	**−0.11 (0.12)**	0.08 (0.30)	**0.16 (0.02)**
	Age (years)	**0.16 (0.02)**	0.07 (0.33)	−0.02 (0.73)
	Race	0.05 (0.45)	−0.02 (0.80)	−0.13 (0.05)
	BMI	0.06 (0.40)	0.02 (0.72)	**0.24 (0.00)**
	Depressive symptoms	0.08 (0.26)	−0.11 (0.12)	−0.13 (0.06)
Aggregated momentary	Intercept	**0.00 (0.01)**	0.00 (0.51)	0.00 (0.57)
	Aggregated momentary loneliness	0.01 (0.84)	0.06 (0.40)	**0.16 (0.03)**
	Age (years)	**0.16 (0.02)**	0.06 (0.42)	−0.05 (0.47)
	Race	0.06 (0.40)	−0.02 (0.82)	−0.12 (0.06)
	BMI	0.06 (0.39)	0.03 (0.71)	**0.25 (0.00)**
	Depressive symptoms	0.04 (0.61)	−0.11 (0.14)	−0.13 (0.07)

*Standardized betas (p) reported; Race was coded as White = 1 and Other = 0; Bold indicates statistical significance at α = 0.05*.

### CRP

Both trait loneliness and aggregated momentary levels of loneliness were significantly associated with higher CRP scores (β = 0.16, *p* = 0.02; β = 0.16, *p* = 0.03, respectively), controlling for the same set of covariates as in the cytokine analyses^4^. Full regression results are presented in Table 4.

### Moderation Effects

Gender and race were tested as moderators between loneliness and each cytokine composite score or CRP, controlling for age, race, BMI, and depressive symptoms. Regression analyses revealed no significant interactions (see Table 5).

**Table 5:** Null results for moderation analyses.

		**Basal composite**	**Stimulated composite**	**CRP**
**Moderation by gender**				
Trait	Intercept	**0.00 (0.02)**	0.00 (0.28)	0.00 (0.90)
	Trait loneliness	**−0.20 (0.03)**	0.17 (0.06)	0.12 (0.16)
	Gender	−0.32 (0.13)	0.39 (0.07)	−0.20 (0.33)
	Age (years)	**0.18 (0.01)**	0.05 (0.44)	−0.02 (0.72)
	Race	0.04 (0.52)	−0.03 (0.72)	−0.11 (0.08)
	BMI	0.05 (0.47)	0.04 (0.58)	**0.24 (0.00)**
	Depressive symptoms	0.07 (0.33)	−0.11 (0.12)	−0.12 (0.09)
	Loneliness × gender	0.35 (0.11)	−0.35 (0.11)	0.12 (0.58)
Aggregated momentary	Intercept	**0.00 (0.01)**	0.00 (0.44)	0.00 (0.40)
	Aggregated momentary loneliness	0.04 (0.71)	0.06 (0.54)	**0.22 (0.02)**
	Gender	0.03 (0.76)	0.06 (0.47)	−0.07 (0.41)
	Age (years)	0.16 (0.03)	0.06 (0.38)	−0.07 (0.31)
	Race	0.06 (0.41)	−0.03 (0.70)	−0.10 (0.15)
	BMI	0.06 (0.37)	0.03 (0.65)	**0.25 (0.00)**
	Depressive symptoms	0.04 (0.63)	−0.11 (0.12)	−0.12 (0.08)
	Loneliness × gender	−0.04 (0.74)	−0.01 (0.95)	−0.10 (0.36)
**Moderation by race**				
Trait	Intercept	**0.00 (0.03)**	0.00 (0.32)	0.00 (0.95)
	Trait loneliness	−0.17 (0.08)	0.13 (0.19)	0.08 (0.39)
	Race	−0.13 (0.54)	0.15 (0.49)	−0.38 (0.06)
	Age (years)	**0.16 (0.02)**	0.06 (0.37)	−0.02 (0.81)
	BMI	0.05 (0.42)	0.02 (0.77)	**0.24 (0.00)**
	Depressive symptoms	0.08 (0.26)	−0.11 (0.12)	**−0.13 (0.06)**
	Loneliness × race	0.20 (0.37)	−0.18 (0.41)	0.27 (0.20)
Aggregated momentary	Intercept	**0.00 (0.01)**	0.00 (0.51)	0.00 (0.57)
	Aggregated momentary loneliness	0.05 (0.60)	0.01 (0.93)	0.13 (0.15)
	Race	0.09 (0.31)	−0.06 (0.50)	−0.14 (0.09)
	Age (years)	**0.15 (0.03)**	0.06 (0.39)	−0.05 (0.49)
	BMI	0.06 (0.36)	0.02 (0.78)	**0.25 (0.00)**
	Depressive symptoms	0.04 (0.62)	−0.11 (0.14)	−0.13 (0.07)
	Loneliness × race	−0.06 (0.57)	0.09 (0.42)	0.03 (0.74)

*Standardized betas (p) reported; Race was coded as White = 1 and Other = 0; Bold indicates statistical significance at α = 0.05*.

## Discussion

Prior research strongly indicates that loneliness is linked to negative health outcomes (Rudatsikira et al., 2007; Meltzer et al., 2013; Holt-Lunstad and Smith, 2016; Rico-Uribe et al., 2018) and suggests that inflammation is one potential mechanism underlying the association between loneliness and health (Hawkley and Cacioppo, 2003; Hawkley et al., 2007; Kiecolt-Glaser et al., 2010; Cacioppo and Cacioppo, 2018). Older adults are not only at greater risk of poor health but have been identified as a sub-population particularly at risk for loneliness (National Academies of Sciences, 2020). Yet, few studies have investigated the link between loneliness and inflammation among individuals over the age of 70 years. The present research helps to fill this gap in the literature, harnessing rich inflammatory data from an ethnically diverse sample to examine cross-sectional associations between loneliness measures and a range of inflammatory markers among adults aged 70–90 years. Whereas, prior research on the association between loneliness and inflammation has utilized trait measures of loneliness, the present research examined linkages with both trait loneliness and aggregated momentary loneliness assessed in daily life. This method enabled, for the first time, examination of whether loneliness reported in daily life is associated with higher inflammation.

We observed significant associations between both loneliness measures (retrospective trait loneliness and aggregated momentary loneliness) and CRP, controlling for age, race, BMI, and depressive symptoms. In contrast, neither loneliness measure was significantly associated with the composite basal cytokine measure or composite stimulated cytokine measure (or, in exploratory analyses, with individual cytokines). Our findings are in contrast to past work linking loneliness to higher cytokine levels during midlife (Nersesian et al., 2018) and past null findings with CRP for adults ages 18–85 years old (for meta-analysis, see: Smith et al., 2020). However, our results are in line with past longitudinal work that has found significant associations between trait loneliness and CRP among different age samples [50–99 years: (Vingeliene et al., 2019) and 50–67 years: (Cole et al., 2007)]. In addition, other psychosocial and behavioral phenomena related to stress (including hostility, insomnia, and childhood adversity) have been associated with CRP but not cytokines (Graham et al., 2006b; Baumeister et al., 2016; Slavish et al., 2018). Compared to cytokines, CRP (an acute phase protein) provides a more temporally stable measure of inflammation and changes less quickly in response to acute stress (Marsland et al., 2017). Therefore, measures of CRP may better capture chronic processes related to psychological stress or illness (Black, 2003; Graham et al., 2006b).

Linkages with CRP are important because both increases in CRP (Ridker, 1998; Danesh, 2000; Libby et al., 2002) and persistently high levels of CRP are risk factors for cardiovascular disease (Ridker, 2003) among middle-aged and older adults. In the present study, average CRP levels for the overall sample (4.30 mg/L) were high [>3 mg/L is considered high risk for cardiovascular disease (Pearson et al., 2003; Ridker, 2003)] and likely reflect the age and racial diversity of the sample (McDade et al., 2006, 2011). Individuals with higher than average trait or aggregated momentary loneliness had higher CRP levels than individuals with lower than average levels of these measures. Taken together, past and current findings suggest that CRP is an important biomarker related to loneliness and health outcomes among older adults.

Interestingly, the retrospective trait loneliness measure and our aggregated momentary measure of loneliness in daily life yielded similar associations with inflammation. To our knowledge, this is the first report of an association between loneliness and a marker of inflammation where loneliness was derived from momentary reports. This is important because the trait and aggregated loneliness measures were only moderately correlated [*r*_(220)_ = 0.46] in the present research, which suggests that they measured different phenomena. Trait assessments of mood are more likely than momentary assessments to be influenced by broad self-perceptions of the self and memory bias (Shiffman et al., 2008). Moreover, levels of momentary loneliness may oscillate in daily life, sometimes depending on environmental or social context (e.g., being at home or alone) (Compernolle et al., 2021). Therefore, each measure offers different information about loneliness. In the present study, both loneliness measures were significantly linked with CRP and not basal or stimulated cytokines.

Neither gender nor race moderated the association between loneliness and inflammation. We expected that associations between loneliness and inflammatory markers might be stronger among women, given previous work indicating that lonelier women tend to exhibit heightened biological responses to stress (Steptoe et al., 2004; Thurston and Kubzansky, 2009). However, prior findings of gender differences in health outcomes related to loneliness have been inconsistent. In addition, we tested moderation by race on an exploratory basis. Previous work has observed higher levels of loneliness (Hawkley et al., 2019) and CRP (McDade et al., 2006, 2011) among Black vs. White older adults, and there are well-established health disparities among Black individuals in diseases linked with CRP (e.g., heart disease, stroke, and diabetes) (Hayward et al., 2000; Wyatt et al., 2003). However, no work to our knowledge has observed race as a moderator of the loneliness-inflammation connection. In the present work, race did not moderate findings, with comparable results observed between Black and White participants (and when comparing Black participants to all others).

## Limitations and Future Directions

There are some limitations in the study that should be considered when interpreting these results. First, our findings are based on cross-sectional data, and as such, the present research cannot be taken to imply causal directionality. Drawing from a stress and health perspective, it is possible that loneliness predicts higher levels of CRP. Conversely, it is also possible that higher levels of CRP predict higher levels of loneliness, with perhaps the influence of general health contributing to both, particularly in late adulthood (Theeke, 2009). From this perspective, it is important to note that CRP values were relatively high in this sample, reflecting our sample of individuals who were recruited to be representative of an older population without regard for health status (individuals aged 70 and older the Bronx, NY); CRP levels in the present research may also reflect our sample including a sizable minority of African-American participants, in whom CRP levels have been observed to be higher than in white participants (Ranjit et al., 2007; Gruenewald et al., 2009; Herd et al., 2012; Ransome et al., 2018). Importantly, in sensitivity analyses, we determined that controlling for health conditions and smoking status did not change findings. Moreover, we controlled for age, race, BMI, and depressive symptoms. As with any study, our results may not generalize beyond our specific sample.

It is also possible that the association between loneliness and inflammation is non-linear such that the association is stronger for those who have higher levels of loneliness. However, average levels of loneliness were generally low in our sample and we were unable to test this hypothesis. In addition, there has been recent interest in making the distinction between social isolation and loneliness to help tease apart the impact of subjective vs. objective experiences. We were not able to address this in the present manuscript, as we did not have a precise measure of social isolation. However, we did have data on whether participants lived alone. Our results did not vary in models that controlled for living alone, suggesting that for our sample the association between loneliness and inflammation was driven more by the perception of feeling alone than by the objective nature of being isolated (results shown in Supplementary Material). Future research to better distinguish between the two concepts (social isolation and loneliness) will be valuable.

Importantly, our regression models controlled for depressive symptoms. Past work has described loneliness and depressive symptoms as being separate, though correlated constructs (Cacioppo et al., 2006a,b). Indeed, depressive symptomatology may very well overlap with loneliness and be related to physical health (although likely not as strongly as clinical depression), which is why it is important to control for depression-related variables in research linking loneliness and inflammation. Our model-building approach for the current study was to choose theoretically meaningful covariates and include them in all models (i.e., stimulated/basal cytokines, CRP, aggregated/trait loneliness) for ease of comparison. In follow-up analyses, we removed depressive symptoms from the models; results were similar but only marginally significant. Although the change in the effect size of loneliness when including depressive symptoms in the models is minimal, this change is likely theoretically meaningful. Partialling depressive symptoms from loneliness should result in the portion of loneliness that is not linked with depressive symptoms (or with how depressive symptoms is measured). It seems likely that loneliness is related to stress-related poorer health in ways that are separate and unique from depressive symptoms. Future research is needed to clarify the associations between loneliness, depressive symptoms, and inflammation.

Another important direction for future studies investigating the connection between loneliness and inflammation will be to better determine specific factors that may explain or modify this connection. As suggested in Smith et al. (2020), mixed findings in the literature linking loneliness and various inflammatory markers could in part be explained by there being indirect pathways between loneliness and inflammation that depend on varied phenomena. Past research showing that loneliness increases immune reactions to biological (Eisenberger et al., 2017) and social stressors (Brown et al., 2018) suggests that loneliness may moderate how the immune system responds to stressors rather than have a direct effect on the immune system. Future research to better determine specific stress-related mechanisms linking loneliness and inflammation would help explain the broad connection between loneliness and health. Lastly, related work among highly lonely samples is needed.

## Conclusion

Our findings contribute to a large evidence-base showing that loneliness is associated with indicators of poor health. Among a racially/ethnically diverse sample of older adults living in the Bronx, New York, both higher trait loneliness and aggregated momentary measures of loneliness were associated with higher levels of CRP, controlling for age, race, BMI, and depressive symptoms. The present findings are the first to our knowledge to link loneliness assessed in daily life with inflammation. Further, this research is among the first to examine the linkage between loneliness and inflammation in a sample comprised solely of older adults, who may be more susceptible to becoming lonely compared to midlife adults due to various life factors related to aging, such as decreases in health and the loss of a partner (Hawkley et al., 2019). Despite our sample reporting low-average levels of loneliness, we detected associations between CRP and two different measures of loneliness. Our findings support the notion that heightened inflammation may be a mechanism underlying the link between loneliness and risk of disease and mortality among older adults. Individuals who evidence larger increases in inflammatory markers in response to the stressors of everyday life may be more vulnerable to chronic systemic inflammation and inflammatory related diseases (Lockwood et al., 2016; Marsland et al., 2017). Thus, future studies using daily and momentary measures of loneliness will be useful to unpack how loneliness and associated stressors in everyday life are associated with inflammation.

## Data Availability Statement

The raw data supporting the conclusions of this article will be made available by the authors, without undue reservation.

## Ethics Statement

This research involving human participants was reviewed and approved by the Albert Einstein College of Medicine Institutional Review Board. Participants provided their written informed consent to participate in this study.

## Author Contributions

All authors listed have made a substantial, direct, and intellectual contribution to the work and approved it for publication.

## Funding

This work was supported in part by National Institute on Aging (NIA) grants RF1 AG056487 (Engeland and Graham-Engeland) and P01AG003949 (Lipton and Sliwinski) and T32 AG049676 to The Pennsylvania State University.

## Conflict of Interest

The authors declare that the research was conducted in the absence of any commercial or financial relationships that could be construed as a potential conflict of interest.

## Publisher's Note

All claims expressed in this article are solely those of the authors and do not necessarily represent those of their affiliated organizations, or those of the publisher, the editors and the reviewers. Any product that may be evaluated in this article, or claim that may be made by its manufacturer, is not guaranteed or endorsed by the publisher.

## Footnotes

^1^This change in protocol was made due to evidence that LPS-stimulated cytokine levels are elevated when collected in heparin-coated compared to EDTA-coated collection tubes. This occurs due to enhanced activation of peripheral blood monocytes. Hence, EDTA tubes are more appropriate for *ex vivo* LPS-stimulation of whole blood. See Heinzelmann and Bosshart (2005).

^2^CRP levels for all individuals were determined from EDTA-coated collection tubes.

^3^In follow-up analyses, structural equation models (SEM) for both stimulated and basal cytokines were estimated using the *lavaan* package in *R*. This approach did not yield different results from the multiple regression models that were conducted with composite cytokine scores. Results are included in Supplemental Material.

^4^Regression models predicting CRP without depressive symptoms as a covariate fell just outside the.05 significance level; trait loneliness: (*trait loneliness models*: with depressive symptoms: β = 0.159, *p* = 0.021, 95% CI = [0.02, 0.24]; without depressive symptoms: β = 0.113, *p* = 0.082, 95% CI = [−0.01, 0.19]; *aggregated momentary loneliness models*: with depressive symptoms: β = 0.155, *p* = 0.027, 95% CI = [0.001, 0.02]; without depressive symptoms: β = 0.111, *p* = 0.093, 95% CI = [−1.72, 3.10]); see Discussion for interpretation.
