# Supplementary material for: The Association Between Loneliness and Inflammation: Findings From an Older Adult Sample
Source: Front Behav Neurosci. 2022 Jan 11;15:801746. doi: 10.3389/fnbeh.2021.801746 (PMC8787084; doi:10.3389/fnbeh.2021.801746)

Supplementary Material

# Table 1. Regression analysis results for the association between loneliness and individual basal cytokines

|  |  | IL-1β | IL-4 | IL-6 | IL-8 | IL-10 | TNF-α |
| --- | --- | --- | --- | --- | --- | --- | --- |
| Trait | Intercept | 0.00 (0.60) | 0.00 (0.89) | 0.00 (0.07) | **0.00 (0.00)** | 0.00 (0.20) | 0.00 (0.36) |
|  | Trait Loneliness | -0.05 (0.53) | 0.00 (0.14) | 0.01 (0.10) | -0.10 (0.15) | -0.10 (0.15) | -0.14 (0.05) |
|  | Age (Years) | 0.05 (0.44) | 0.00 (0.36) | **0.18 (0.01)** | -0.03 (0.66) | **0.21 (0.00)** | **0.16 (0.02)** |
|  | Race | -0.05 (0.46) | 0.00 (0.69) | 0.01 (0.92) | 0.06 (0.41) | **0.13 (0.04)** | 0.10 (0.14) |
|  | BMI | 0.01 (0.85) | 0.00 (0.96) | **0.21 (0.00)** | -0.02 (0.72) | -0.10 (0.11) | **0.14 (0.04)** |
|  | Depressive symptoms | 0.05 (0.45) | 0.00 (0.75) | 0.03 (0.69) | -0.02 (0.82) | 0.10 (0.16) | **0.17 (0.02)** |
| Aggregated Momentary | Intercept | 0.00 (0.72) | 0.00 (0.97) | 0.00 (0.07) | **0.00 (0.00)** | 0.00 (0.13) | 0.00 (0.44) |
|  | Aggregated Momentary Loneliness | -0.04 (0.58) | 0.00 (0.83) | 0.12 (0.10) | -0.02 (0.80) | -0.03 (0.72) | 0.07 (0.36) |
|  | Age (Years) | 0.06 (0.39) | 0.00 (0.38) | **0.18 (0.01)** | -0.04 (0.53) | **0.22 (0.00)** | **0.15 (0.03)** |
|  | Race | -0.05 (0.45) | 0.00 (0.76) | 0.01 (0.92) | 0.05 (0.43) | **0.14 (0.04)** | 0.11 (0.10) |
|  | BMI | 0.01 (0.87) | 0.00 (0.94) | **0.21 (0.00)** | -0.03 (0.70) | -0.10 (0.12) | **0.14 (0.03)** |
|  | Depressive symptoms | 0.05 (0.47) | 0.00 (0.36) | 0.03 (0.69) | -0.03 (0.69) | 0.07 (0.31) | 0.09 (0.18) |

*Note.* Standardized betas (*p*) reported; Race was coded as White=1/Other=0; Bold indicates statistical significance at α = 0.05

# Table 2. Regression analysis results for the association between loneliness and individual stimulated cytokines

|  |  | IL-1β | IL-4 | IL-6 | IL-8 | IL-10 | TNF-α |
| --- | --- | --- | --- | --- | --- | --- | --- |
| Trait | Intercept | 0.00 (0.33) | 0.00 (0.71) | 0.00 (0.17) | **0.00 (0.00)** | 0.00 (0.59) | **0.00 (0.00)** |
|  | Trait Loneliness | 0.01 (0.85) | 0.08 (0.26) | 0.06 (0.42) | -0.10 (0.15) | 0.07 (0.34) | 0.06 (0.37) |
|  | Age (Years) | 0.09 (0.18) | 0.09 (0.19) | 0.09 (0.19) | -0.03 (0.66) | 0.03 (0.61) | 0.01 (0.91) |
|  | Race | 0.06 (0.35) | -0.04 (0.54) | -0.11 (0.09) | 0.06 (0.41) | 0.05 (0.45) | -0.04 (0.54) |
|  | BMI | -0.02 (0.76) | 0.05 (0.48) | 0.05 (0.42) | -0.02 (0.72) | -0.00 (0.98) | 0.04 (0.52) |
|  | Depressive symptoms | -0.10 (0.19) | **-0.15 (0.04)** | -0.10 (0.15) | -0.02 (0.82) | -0.09 (0.23) | -0.04 (0.62) |
| Aggregated Momentary | Intercept | 0.00 (0.23) | 0.00 (0.79) | 0.00 (0.11) | **0.00 (0.00)** | 0.00 (0.39) | **0.00 (0.00)** |
|  | Aggregated Momentary Loneliness | 0.09 (0.20) | -0.03 (0.67) | 0.05 (0.48) | -0.07 (0.33) | 0.09 (0.21) | 0.07 (0.37) |
|  | Age (Years) | 0.08 (0.26) | 0.09 (0.18) | 0.08 (0.24) | -0.02 (0.79) | 0.02 (0.77) | -0.00 (0.96) |
|  | Race | 0.07 (0.30) | -0.05 (0.49) | -0.12 (0.10) | 0.05 (0.43) | 0.06 (0.41) | -0.04 (0.57) |
|  | BMI | -0.02 (0.80) | 0.05 (0.50) | 0.06 (0.41) | -0.03 (0.70) | 0.00 (0.98) | 0.05 (0.50) |
|  | Depressive symptoms | -0.12 (0.09) | -0.11 (0.12) | -0.10 (0.16) | -0.03 (0.69) | -0.09 (0.19) | -0.04 (0.63) |

*Note.* Standardized betas (*p*) reported; Race was coded as White=1/Other=0; Bold indicates statistical significance at α = 0.05

# Table 3. Regression analysis results for main effects models, controlling for ‘living alone’ status

|  |  | **Basal Composite** | **Stimulated Composite** | **CRP** |
| --- | --- | --- | --- | --- |
|  | Intercept | **0.00 (0.03)** | 0.00 (0.36) | 0.00 (0.98) |
| **Trait** | Trait Loneliness | -0.12 (0.10) | 0.08 (0.30) | **0.15 (0.03)** |
|  | Age (Years) | **0.15 (0.03)** | 0.07 (0.34) | -0.03 (0.60) |
|  | Race | 0.05 (0.47) | -0.02 (0.80) | **-0.13 (0.04)** |
|  | BMI | 0.05 (0.44) | 0.02 (0.73) | **0.24 (0.00)** |
|  | Depressive symptoms | 0.09 (0.21) | -0.11 (0.12) | -0.12 (0.06) |
|  | Living alone | 0.07 (0.30) | 0.00 (0.10) | 0.11 (0.10) |
| **Aggregated Momentary** | Intercept | **0.00 (0.01)** | 0.00 (0.51) | 0.00 (0.53) |
|  | Aggregated Momentary Loneliness | 0.01 (0.90) | 0.06 (0.41) | **0.15 (0.04)** |
|  | Age (Years) | 0.15 (0.03) | 0.06 (0.42) | -0.06 (0.38) |
|  | Race | 0.05 (0.42) | -0.02 (0.82) | -0.13 (0.05) |
|  | BMI | 0.05 (0.39) | 0.03 (0.71) | **0.25 (0.00)** |
|  | Depressive symptoms | 0.04 (0.54) | -0.11 (0.14) | -0.11 (0.10) |
|  | Living alone | 0.06 (0.39) | 0.00 (0.98) | 0.11 (0.09) |

*Note.* Standardized betas (*p*) reported; Race was coded as White=1/Other=0; Living alone was coded as Yes=1/No=0; Bold indicates statistical significance at α = 0.05

# Figure 1. Scatterplot for multiple regression model, trait loneliness predicting basal cytokine composite


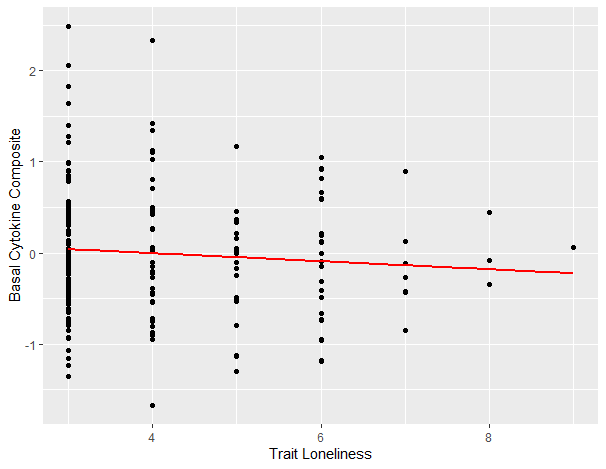


# Figure 2. Scatterplot for multiple regression model, trait loneliness predicting stimulated cytokine composite


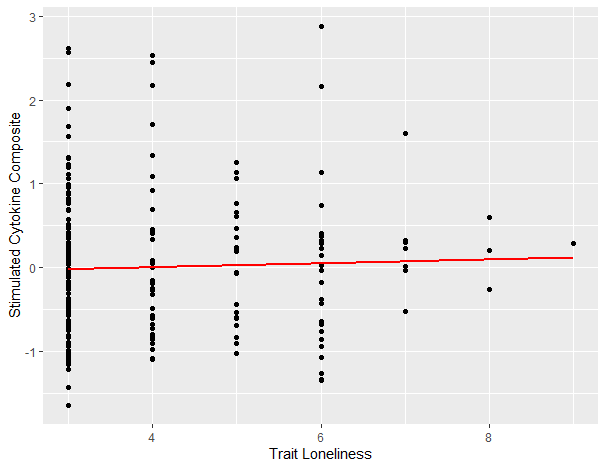


# Figure 3. Scatterplot for multiple regression model, trait loneliness predicting CRP


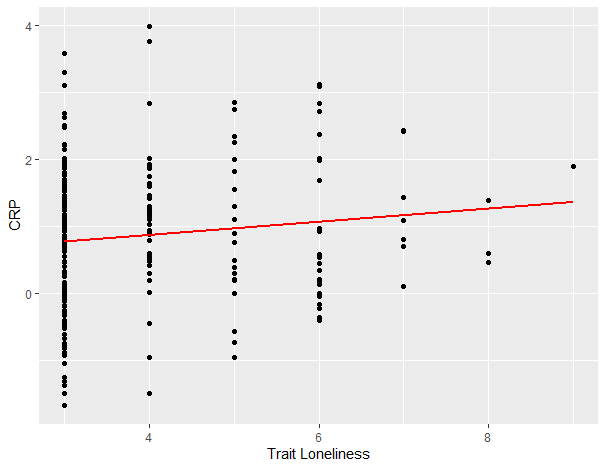


# Figure 4. Scatterplot for multiple regression model, aggregated momentary loneliness predicting basal cytokine composite

**
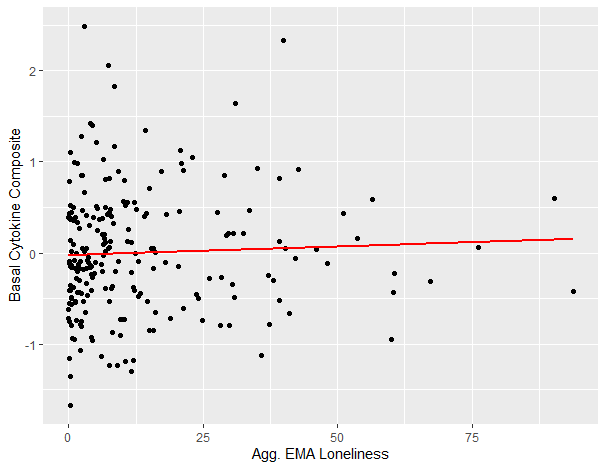
**

# Figure 5. Scatterplot for multiple regression model, aggregated momentary loneliness predicting stimulated cytokine composite

**
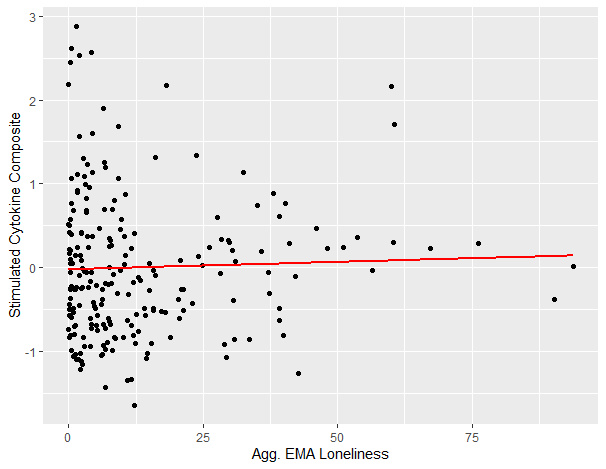
**

# Figure 6. Scatterplot for multiple regression model, aggregated momentary loneliness predicting CRP


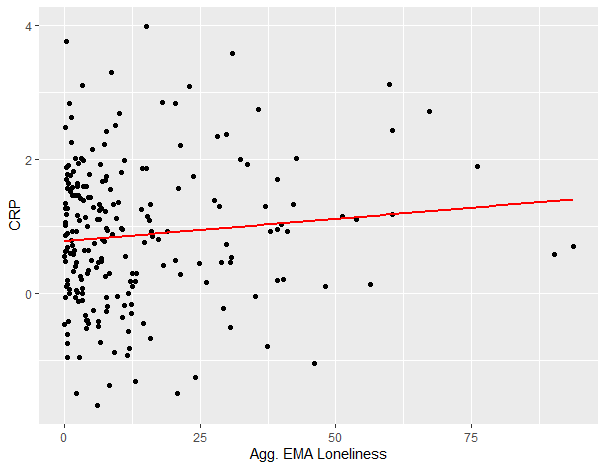


# Figure 7. Trait loneliness predicting latent basal cytokine composite variable


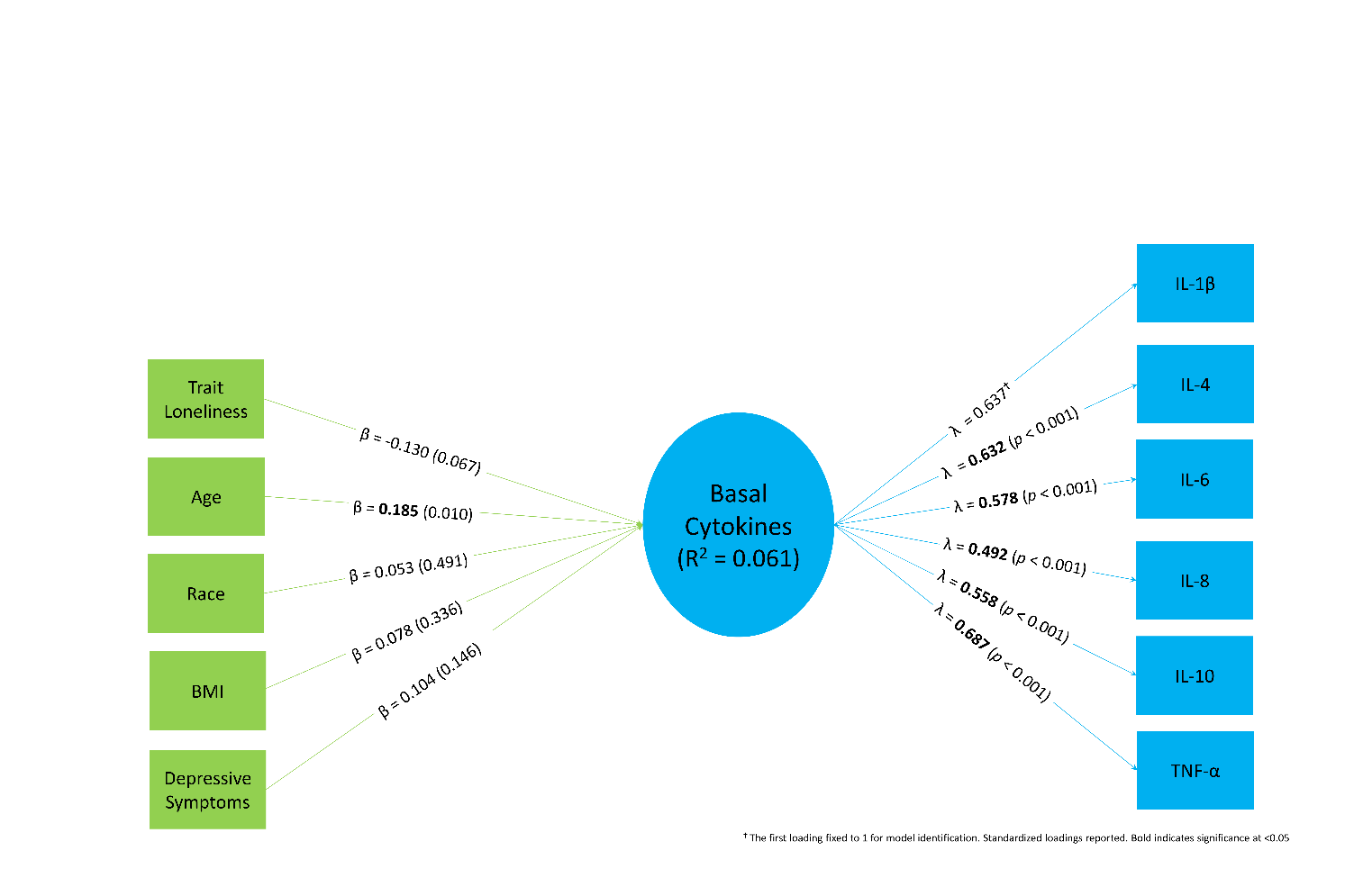


# Figure 8. Trait loneliness predicting latent stimulated cytokine composite variable


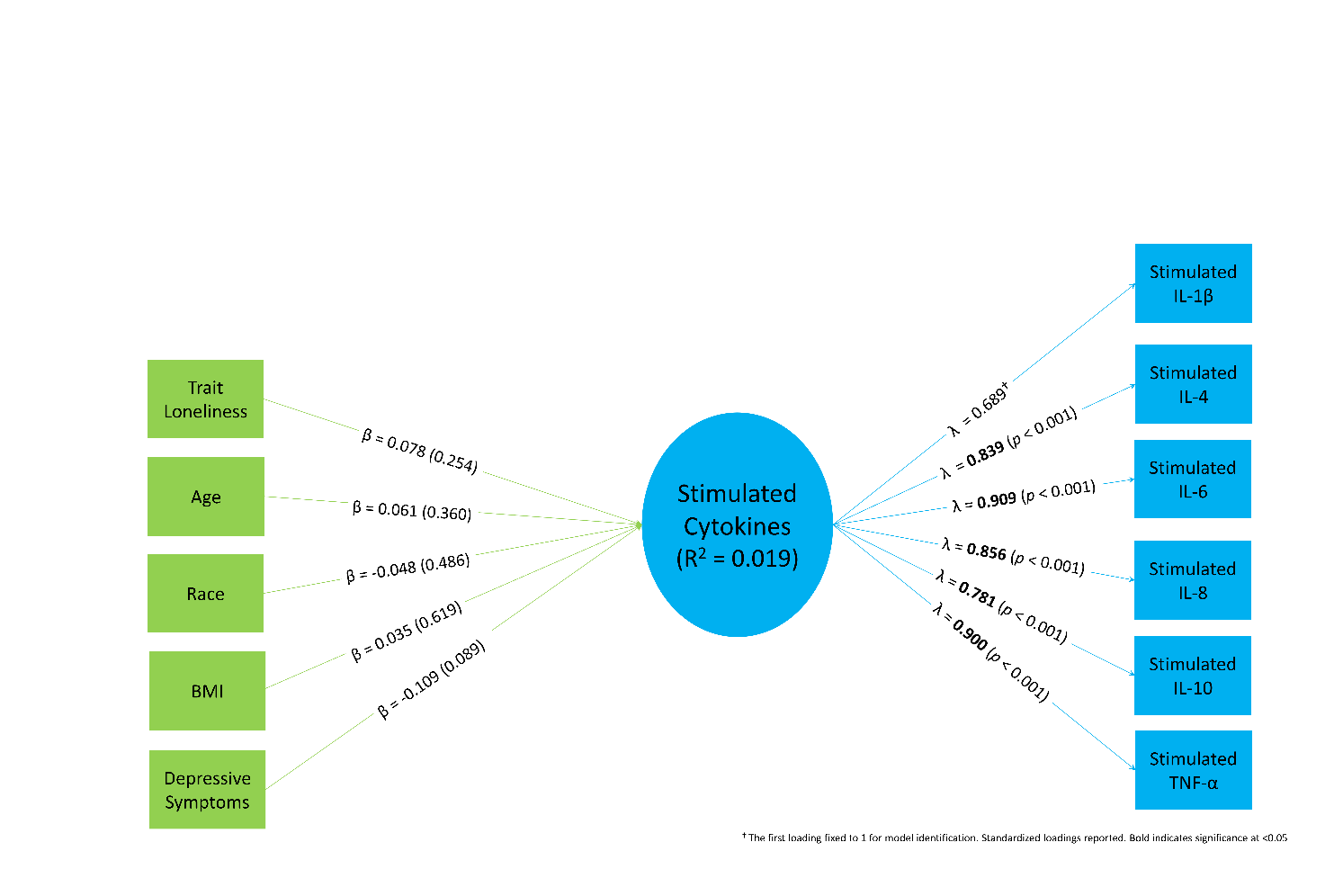


# Figure 9. Aggregated momentary loneliness predicting latent basal cytokine composite variable


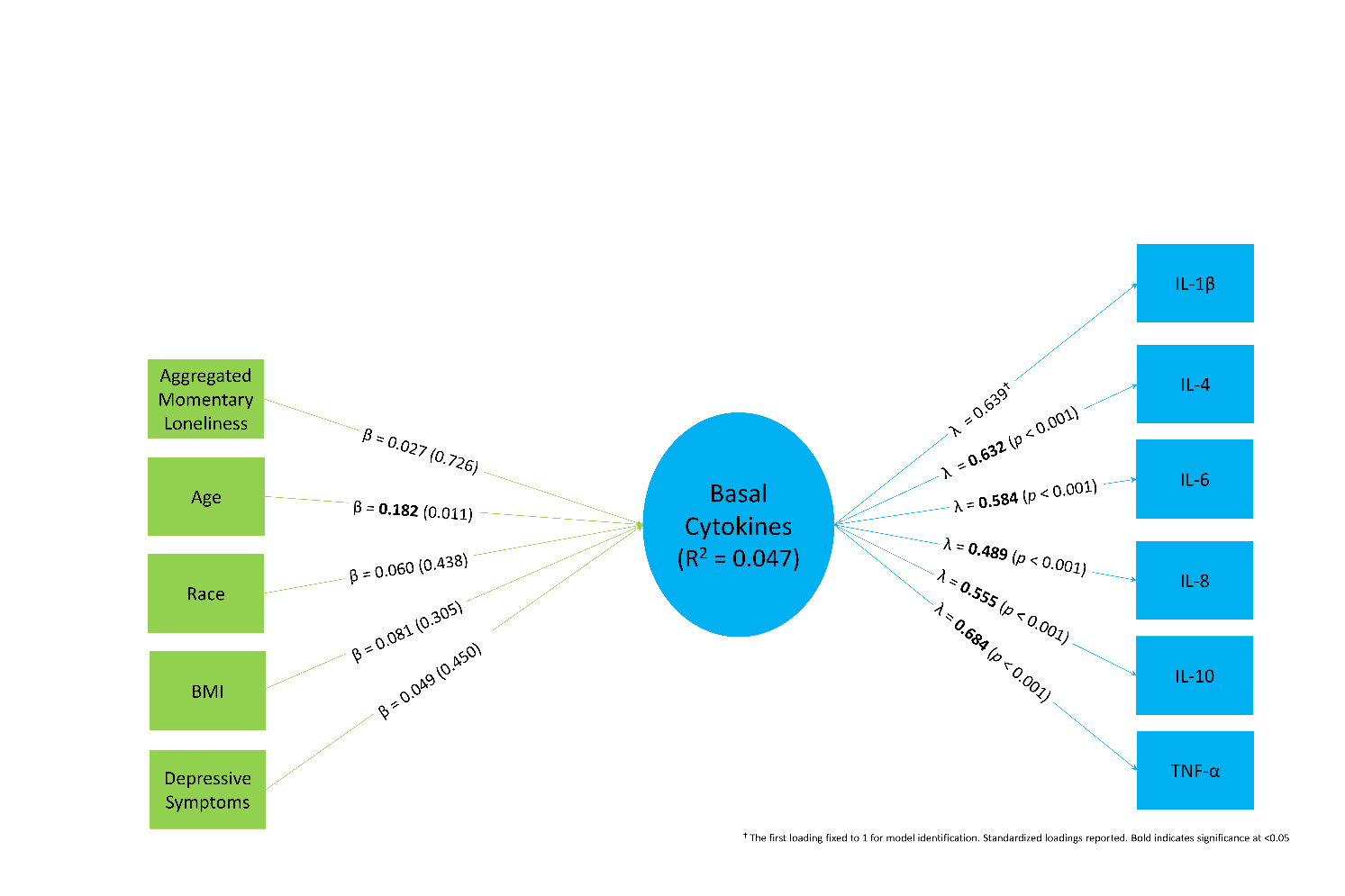


# Figure 10. Aggregated momentary loneliness predicting latent stimulated cytokine composite variable


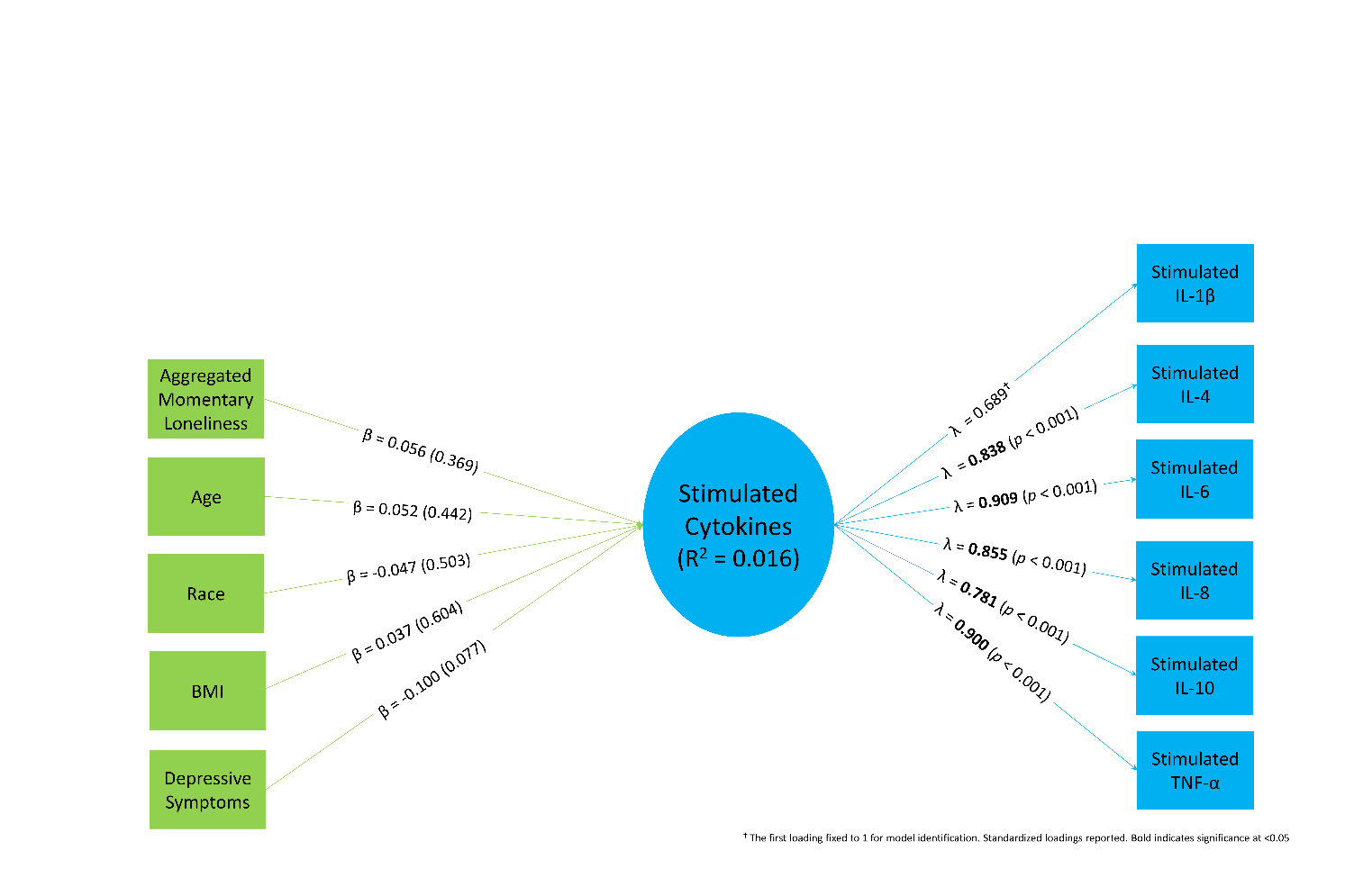

Supplement: Supplementary file 1 [file Data_Sheet_1.docx]
